# Hepatoprotection by Traditional Essence of Ginseng against Carbon Tetrachloride—Induced Liver Damage

**DOI:** 10.3390/nu12103214

**Published:** 2020-10-21

**Authors:** Yi-Ju Hsu, Chao-Yun Wang, Mon-Chien Lee, Chi-Chang Huang

**Affiliations:** Graduate Institute of Sports Science, National Taiwan Sport University, Taoyuan 33301, Taiwan; ruby780202@ntsu.edu.tw (Y.-J.H.); 1080213@ntsu.edu.tw (C.-Y.W.); 1061304@ntsu.edu.tw (M.-C.L.)

**Keywords:** ginsenosides, hepatoprotection, liver injury, anti-oxidant

## Abstract

The peroxide produced in the lipid metabolic process attacks liver cells and causes liver injury. Ginsenosides have been shown to have anti-oxidation abilities and to mend myocardial damage. This study evaluated the effect of traditional ginseng essence (TEG) in preventing chemical liver damage induced by carbon tetrachloride (CCl_4_). Forty 8-week-old male Sprague Dawley (SD) rats were divided into five groups: control, liver injury (CCl_4_), and TEG by oral gavage at 0.074, 0.149, or 0.298 g/kg/day for nine weeks. Liver injury biochemical indicators, antioxidant enzyme activity, and lipid contents in liver tissues were evaluated. The liver appearance was observed, and histopathological tests were conducted to estimate whether TEG-antagonized oxidants further ameliorated liver injury. The results show that, after supplementation of TEG for nine consecutive weeks and CCl_4_—induced liver injury for eight weeks, the levels of liver injury biochemical indicators in animal serum decreased significantly, and, in liver tissue, antioxidant activity was significantly improved and accumulation of lipids was decreased. Pathological sections exhibited reduced liver lipid accumulation and fibrosis. As discussed above, TEG can increase the antioxidant capacity in the liver and the maintenance of hepatocyte function, protecting the liver from chemical injury and improving healthcare.

## 1. Introduction

An irregular lifestyle may cause abnormal lipid metabolism in the body. Converting lipophilic xenobiotics to hydrophilic forms leads to incomplete pharmacological or biological activity and conversion effects. The harmful reactive intermediates produced, such as free radicals and redox-active reactants, can induce metabolic pressure [1]. Metabolic pressure or an inflammatory response may damage liver cells and even cause steatosis and cirrhosis [2]. In hepatic stellate cells, collagen synthesis, which plays a direct causative role in liver fibrogenesis, is triggered by lipid peroxidation caused by oxidative stress [3]. Above all, liver cell damage is closely correlated to oxidative stress.

A CCl_4_—induced liver injury model has been used to evaluate the chemical liver injury. The mechanism occurs during liver metabolism, wherein cytochrome P450 (CYP) enzymes in CCl_4_ form the trichloromethyl radical (CCl_3_) [4]. This process impairs crucial cellular processes and induces extensive cell damage and apoptosis. In hepatic apoptosis and fibrosis, the synthesis of cellular phospholipids refers to the incorporation of phospholipids into lipoproteins, leading to the accumulation of triglycerides [5].

Ginseng is one of the few medicinal plants that can be eaten from root to leaf. The active ingredients of ginseng are more than 30 ginsenosides. These ginsenosides can be classified into two main groups: glycosides of 20(S)-protopanaxadiol (Rb1, Rb2, Rc, Rd, Rg3, and Rh2) and glycosides of 20(S)-protopanaxatriol (Re, Rf, Rg1, Rg2, Rh1, and R1) [6]. *Panax ginseng* (G-115) and *P. quinquefolius* (CNT-2000) are the most widely used breeds in medicinal products [7]. One study used high-performance liquid chromatography (HPLC) to analyze these two different breeds of ginseng and found that the major ingredients of *P. ginseng* are Rb1, Rb2, Rc, Rg1, and Re, and that *P. quinquefolius* has higher concentrations of Rb1 and Re [8]. These medicinal ingredients have multiple biotransformation effects. They can reduce inflammation and oxidative stress [9]. Another study showed that Tibetan *Rhodiola rosea* L can reduce oxidative stress and protect hepatic cells [10]. In particular, in one previous study, ginsenoside Rg2 was shown to modulate the protein p53 in cell apoptosis and, thereby, improve vascular dementia or other ischemic injury [11]. The antioxidant mechanism of ginsenosides may reduce oxidative stress and effectively improve liver injury. In this study, we investigated the effects of different doses of traditional essence of ginseng (TEG) supplements on the liver cell and functional damage after CCl_4_—induced liver injury in rats.

## 2. Materials and Methods

### 2.1. Materials

Traditional essence of ginseng (TEG) derived from *P. ginseng* (Asian ginseng) and *P. quinquefolius* (American ginseng) manufactured with good manufacturing practices (GMP) was purchased from LAO XIE ZHEN Co., Ltd. (Taipei City, Taiwan) and stored at −20 °C for the following experiments. The dosage of TEG for rats was calculated based on a human with a body weight of 60 kg. The metabolic conversion based on body area for the rats was 6.2, and the 1X daily-recommended dose was calculated as follows: 1.44 (g)/60 (kg) = 0.024 × 6.2 = a rat dose of 0.149 g/kg. This formula is in line with guidelines from the US Food and Drug Administration.

The content of the TEG, which included ginsenoside Rg2, was determined by a high-performance liquid chromatography (HPLC) method described previously [12] with some modification. A Waters 600 pump system with a 717 plus HPLC autosampler and a Waters 2996 tunable absorbance detector were used to analyze the ginsenoside Rg2 in a Cosmosil 5C_18_-MS-II column (i.d. 4.6 × 250 mm) at 203 nm. A mixture of acetonitrile and H_2_O/potassium dihydrogen phosphate of 1000 mL:2.72 g was used as the mobile phase at a flow rate of 1 mL/min and an injection volume of 20 μL.

### 2.2. Animals and Treatment

Male eight-week-old Sprague Dawley (SD) rats (BioLASCO, Yi-Lan, Taiwan) weighing around 250 g were housed within cages at 22 ± 2 °C and 60% ± 10% relative humidity with a 12 h dark/light cycle, with ad libitum access to food (No. 5001, PMI Nutrition International, Brentwood, MO, USA) and reverse osmosis water. The experimental protocol was approved by the Animal Care and Use Committee (IACUC) No. 10,805 for the ethical use of animals in experiments at the National Taiwan Sport University.

**Figure 1:**
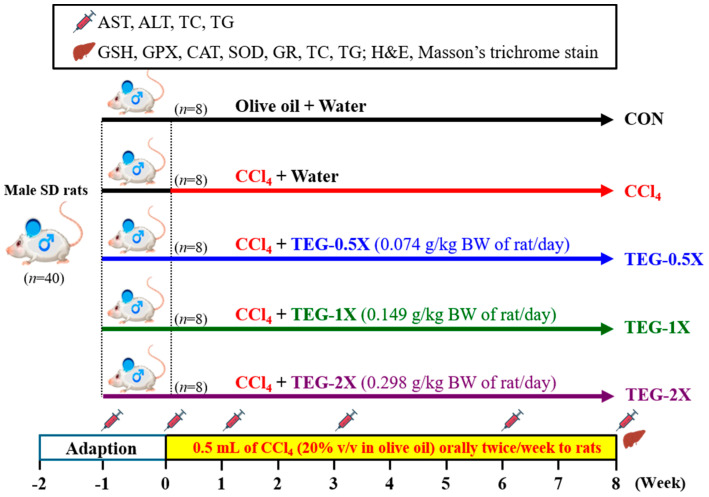
Experimental design. Forty Sprague Dawley (SD) rats were randomized into five groups (*n* = 8 per group): olive oil (CON), carbon tetrachloride (CCl_4_) per oral (p.o.), and dietary supplementation with a traditional essence of ginseng (TEG) doses of 0.074, 0.149, and 0.289 mg/kg body weight (BW) plus CCl_4_ (TEG-0.5X, TEG-1X, and TEG-2X, respectively). CON: Healthy rats orally received a volume of water equivalent to body weight (BW). The CCl_4_, TEG-0.5X, TEG-1X, and TEG-2X groups were orally administrated with CCl_4_ dissolved in olive oil twice a week (20% CCl_4_ in olive oil, 0.5 mL/rat), while the CON group was orally administrated with olive oil only (0.5 mL/rat). CON, control group. CCl_4_, CCl_4_ administration only. TEG-0.5X, CCl_4_ administration with 0.5 times the daily recommended dosage of the TEG. TEG-1X, CCl_4_ administration with daily recommended dosage of the TEG. TEG-2X, CCl_4_ administration with 2 times the daily recommended dosage of the TEG. Liver (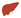), blood (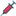). AST: aspartate transaminase. ALT: alanine transaminase. TC: total cholesterol. TG: triglyceride. GSH: glutathione. GPX: glutathione peroxidase. SOD: superoxide dismutase. CAT: catalase. GR: glutathione reductase.

Forty SD rats were randomized into five groups (*n* = 8 per group): olive oil (CON), CCl_4_ per oral (p.o.), and dietary supplementation with TEG doses of 0.074, 0.149, and 0.289 mg/kg body weight (BW) plus CCl_4_ (TEG-0.5X, TEG-1X, and TEG-2X, respectively). The detailed experimental design is illustrated in Figure 1. After 1 week of olive oil (CON and CCl_4_ groups) or TEG treatment, the CCl_4_, TEG-0.5X, TEG-1X, and TEG-2X groups were orally administrated with CCl_4_ dissolved in olive oil twice a week (20% CCl_4_ in olive oil, 0.5 mL/rat) while the control group (CON) group was orally administrated with olive oil only (0.5 mL/rat). Moreover, the 0.5X, 1X, and 2X groups were continuously treated with TEG, whereas the CON and CCl_4_ groups were treated with olive oil every day. TEG was treated after 1 h of CCl_4_ administration to avoid the interaction with CCl_4_ [13]. The purchase and storage of CCl_4_ followed the Chemical Management System of National Taiwan Sport University for toxic chemical substances. The food and water intake were monitored daily and the rats were weighed to determine their body weight every week.

### 2.3. Clinical Biochemical and Hematological Profile Assay

The rats fasted overnight for 8–12 h before blood was collected. Four hours after the ginseng supplement was consumed, blood samples were drawn from the tail veins of the rats. At the end of the experiment (eighth week), the rats were sacrificed by 95% CO_2_ exposure and their blood and organs were collected for analysis. The collected blood samples were centrifuged at 4500 rpm for 15 min at 4 °C. Serum biochemical analyses of aspartate transaminase (AST) and alanine transaminase (ALT) activities, albumin, and total cholesterol (TC) and triacylglycerol (TG) levels were conducted with a 7150 Automatic blood chemistry analyzer (Hitachi Co., Ltd., Tokyo, Japan).

### 2.4. Hepatic Antioxidant Levels

Liver tissue samples of 30 mg were collected and rinsed in 5–10 mL of cold buffer (i.e., phosphate-buffered saline) and centrifuged at 10,000× *g* for 15 min at 4 °C to remove blood cells and clots for a subsequent test. Biochemical kits (Cayman Chemical Co., Ann Arbor, MI, USA) were used to determine the hepatic reduced glutathione (GSH) concentration, glutathione peroxidase (GPX), glutathione reductase (GR), superoxide dismutase (SOD), and catalase (CAT) activities.

### 2.5. Hepatic Lipid Profile Assay

Liver tissues (350 mg) were homogenized in 2 mL of cold buffer (i.e., chloroform/isopropanol/NP40 = 7:11:0.1) and the samples were centrifuged at 10,000× *g* for 10 min at 4 °C for analysis. After removal of the supernatants, samples were remixed with a diluting buffer (50 mM sodium phosphate, pH 7.2). The remixed hepatic triacylglycerol (TG) was determined using a triacylglycerol fluorometric assay kit (item number: 10010303, Cayman Chemical Co., MI, USA).

The total cholesterol (TC) concentration was determined using a cholesterol fluorometric assay kit (item number: 10007640, Cayman Chemical Co., MI, USA). For this test, 100 μL of samples were added to wells, and the plate cover was removed to initiate the reactions by adding 50 μL of a prepared assay cocktail to all of the wells. Liver extractions were mixed well with the reagent buffer and the absorbance value was measured under optical density (OD) of 560 nm.

### 2.6. Pathological Examination of Liver Tissues

After being cleaned with saline, the liver tissue was collected immediately and maintained at −80 °C until analysis. One sample of liver tissue (1 cm × 1 cm) was cut from the largest right lobe and fixed in 40 g/L formaldehyde solution for histology. Hematoxylin–eosin dye (H&E stain) and Masson’s trichrome were used to stain the liver tissue for histological examinations.

The H&E stain was used to evaluate chronic liver damage, including hepatocyte gross necrosis, fatty change, and fibrosis. Levels of steatosis and inflammatory cell infiltration were assessed by semi-quantitative histological evaluation. The scale of liver damage ranged from 0 to 4, where 0 = absent, 1 = trace, 2 = mild, 3 = moderate, and 4 = severe [14]. Masson’s trichrome stain was used to evaluate collagenous fibers.

### 2.7. Statistics Analysis

Values are presented as mean ± SD. To evaluate differences between groups, one-way analysis of variance (ANOVA) was used. A Cochran—Armitage test with SAS 9.0 software (SAS Inst., Cary, NC, USA) was used to estimate the dose effect with *p*-values of less than 0.05 indicating a statistical significance.

## 3. Results

### 3.1. Content of Ginsenoside Rg2 in TEG

**Figure 2:**
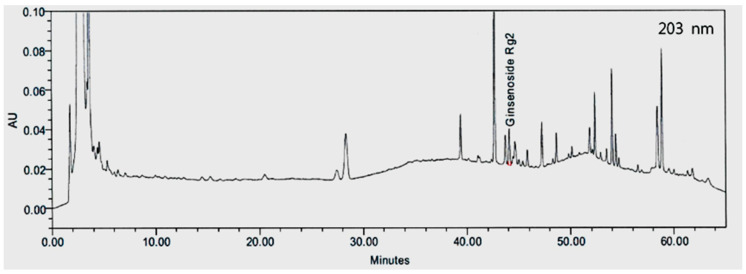
High-performance of liquid chromatography (HPLC) chromatogram of ginsenoside Rg2 in traditional essence of ginseng (TEG).

The retention time of ginsenoside Rg2 was 44.1 min (Figure 2). Based on the calibration curve, the content of total ginsenoside Rg2 in the TEG was 0.88 mg/g.

### 3.2. Effects of TEG on Blood Parameters in Rats with CCl_4_—Induced Liver Damage

**Figure 3:**
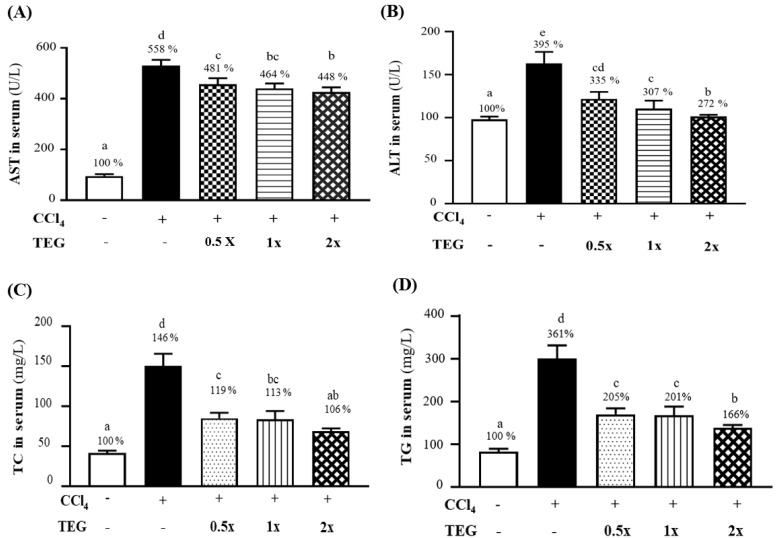
Effect of traditional essence of ginseng (TEG) supplementation on blood parameters in (**A**) aspartate transaminase (AST), (**B**) alanine transaminase (ALT), (**C**) total cholesterol (TC), and (**D**) triglyceride (TG) with carbon tetrachloride (CCl_4_)—induced liver damage at the eighth week. Data are presented as mean ± SD for *n* = 8 rats in each group. Different letters (a, b, c, d, e) mean significant difference at *p* < 0.01 according to one-way ANOVA.

Aspartate transaminase (AST) and alanine transaminase (ALT) activities are liver function markers. After nine weeks of supplementation with TEG and eight weeks of CCl_4_—induced liver injury, the changes in the liver function biochemical index in the blood of rats in each group were examined. The AST activities in the serum of the CON, CCl_4_, TEG-0.5X, TEG-1X, and TEG-2X groups were 95 ± 8, 530 ± 23, 457 ± 24, 441 ± 14, and 426 ± 19 (U/L), respectively. The 1X and TEG-2X groups supplemented with TEG exhibited dose trends. The AST activities were significantly lower, by approximately 13.83%, 16.95%, and 19.75% in these groups, respectively, relative to the CCl_4_ group (Figure 3A) (*p* < 0.0001). The changes in the serum ALT activities in the CON, CCl_4_, TEG-0.5X, TEG-1X, and TEG-2X groups were 43 ± 7, 170 ± 15, 144 ± 19, 132 ± 16, and 117 ± 12 (U/L), respectively. The ALT activities of the TEG-0.5X, TEG-1X, and TEG-2X groups exhibited dose trends, which were significantly lower than in the CCl_4_ group by about 15.29% (*p* = 0.0008), 22.35%, and 31.18% (relative to the CCl_4_ group), respectively (Figure 3B) (*p* < 0.0001). Statistical analysis showed no significant differences in albumin concentration among the five groups.

Concentrations of plasma total cholesterol (TC) and triglyceride (TG) have a high correlation with chronic liver disease [15]. The serum TC concentrations in the CON, CCl_4_, TEG-0.5X, TEG-1X, and TEG-2X groups were 68 ± 4, 99 ± 6, 81 ± 7, 77 ± 8, and 72 ± 7 (mg/dL), respectively, which is significantly lower, by 27%, 33%, and 40%, when compared to the CCl_4_ group (*p* < 0.0001). The TG concentrations in the CON, CCl_4_, TEG-0.5X, TEG-1X, and TEG-2X groups were 83 ± 6, 300 ± 31, 170 ± 14, 167 ± 21, and 138 ± 7 (mg/dL), respectively, which is significantly lower, by 43.33%, 44.38%, and 53.88% in the TEG-0.5X, TEG-1X, and TEG-2X groups, respectively, than in the CCl_4_ group (Figure 3C) (*p* < 0.0001).

The intake of CCl_4_ was the main reason for the increase in the liver AST, ALT, TC, and TG contents of the animals, and the supplementation of 0.5X, 1X, and 2X doses of TEG for nine weeks effectively reduced the effects of increased liver AST, ALT, TC, and TG contents caused by CCl_4_—induced liver injury.

### 3.3. TEG Effects on Hepatic Antioxidative Parameters in Rats with CCl_4_—Induced Liver Damage

Table 1 shows the activities of GSH, GPX, GR, SOD, and CAT with CCl_4_—induced liver damage at the eighth week. The liver GSH content in the CCl_4_ group was significantly reduced by 10.56% (*p* = 0.0004) after 8 weeks of CCl_4_—induced liver injury compared with the CON group. However, in the TEG-0.5X, TEG-1X, and TEG-2X groups, the GSH contents were significantly increased about 1.12-fold (*p* = 0.0004), 1.11-fold (*p* = 0.0006), and 1.13-fold compared with the CCl_4_ group (*p* = 0.0001). GPX activity in the TEG-0.5X, TEG-1X, and TEG-2X groups significantly increased about 1.09-fold (*p* = 0.0003), 1.10-fold (*p* = 0.0002), and 1.25-fold above that of the CCl_4_ group (*p* < 0.0001). GR activity of the CON group had no significant difference with the TEG-0.5X and TEG-1X groups. In these groups, the GR activity was about 1.53-fold higher than that of the CCl_4_ group (*p* < 0.0001). Additionally, in the TEG-2X group, it was significantly higher by about 1.61-fold (*p* < 0.0001) than that in the CCl_4_ group, and this group had even higher activity than the other four groups. Hepatic SOD activity in the CON and TEG-2X groups was significantly increased about 1.20-fold when compared with the CCl_4_ group (*p* = 0.0027). Moreover, CAT activity in the TEG-0.5X, TEG-1X, and TEG-2X groups was about 1.08-fold to 1.12-fold higher than that in the CCl_4_ group. The TEG-2X group had the highest level among the TEG groups. It was nearly equal to that of the CON group.

### 3.4. Effects of TEG on Hepatic Lipid Profiles in Rats with CCl_4_—Induced Liver Damage

**Figure 4:**
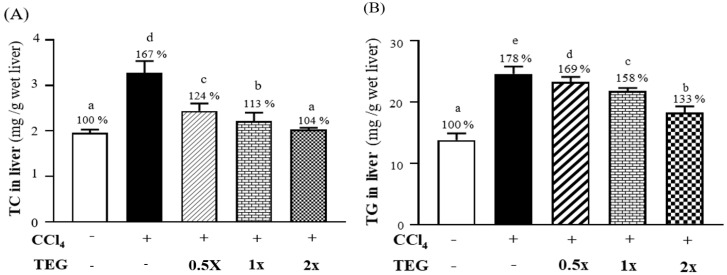
Effect of traditional essence of ginseng (TEG) supplementation on blood parameters: (**A**) total cholesterol (TC) in liver contents and (**B**) triglyceride (TG) in liver contents with carbon tetrachloride (CCl_4_)—induced liver damage at the eighth week. Data are presented as mean ± SD for *n* = 8 rats in each group. Different letters (a, b, c, d, e) mean significant difference at *p* < 0.01 according to one-way ANOVA.

The changes in the hepatic TC content in the CON, CCl_4_, TEG-0.5X, TEG-1X, and TEG-2X groups were 1.96 ± 0.07, 3.27 ± 0.26, 2.44 ± 0.16, 2.21 ± 0.19, and 2.03 ± 0.04 (mg/g wet liver), respectively. The hepatic TC contents of the TEG-0.5X, TEG-1X, and TEG-2X groups were significantly lower, by approximately 25.42%, 32.38%, and 37.81%, respectively, than in the CCl_4_ group (*p* < 0.0001) (Figure 4A). The hepatic TG levels in the CON, CCl_4_, TEG-0.5X, TEG-1X, and TEG-2X groups were 13.8 ± 1.1, 24.6 ± 1.2, 23.3 ± 0.8, 21.8 ± 0.5, and 18.3 ± 1.0 (mg/g wet liver), respectively. In the TEG-0.5X, TEG-1X, and TEG-2X groups, hepatic TC contents were significantly lower, by about 5.36% (*p* = 0.0077), 11.52% (*p* < 0.0001), and 25.65%, respectively, than in the CCl_4_ group (*p* < 0.0001) (Figure 4B).

### 3.5. Effects of TEG on Weight of Liver Changes in Rats with CCl_4_—Induced Liver Damage

Changes in the relative liver weights of the animals in the CON, CCl_4_, TEG-0.5X, TEG-1X, and TEG-2X groups were 2.80 ± 0.36, 3.62 ± 0.56, 3.57 ± 0.47, 3.28 ± 0.38, and 2.98 ± 0.29 (%), respectively. Statistical analysis showed that the relative liver weights of the CCl_4_, TEG-0.5X, and TEG-1X groups increased by 1.29-fold (*p* = 0.0007), 1.27-fold, (*p* = 0.0011) and 1.17-fold (*p* = 0.0295), respectively, after 8 weeks of CCl_4_—induced liver injury. The relative liver weight of the TEG-2X group was significantly lower, by about 17.49%, relative to that of the CCl_4_ group (*p* = 0.0072) (Table 2).

### 3.6. Subacute Histopathology Evaluation and Effect of TEG against CCl_4_—Induced Hepatotoxicity

**Figure 5:**
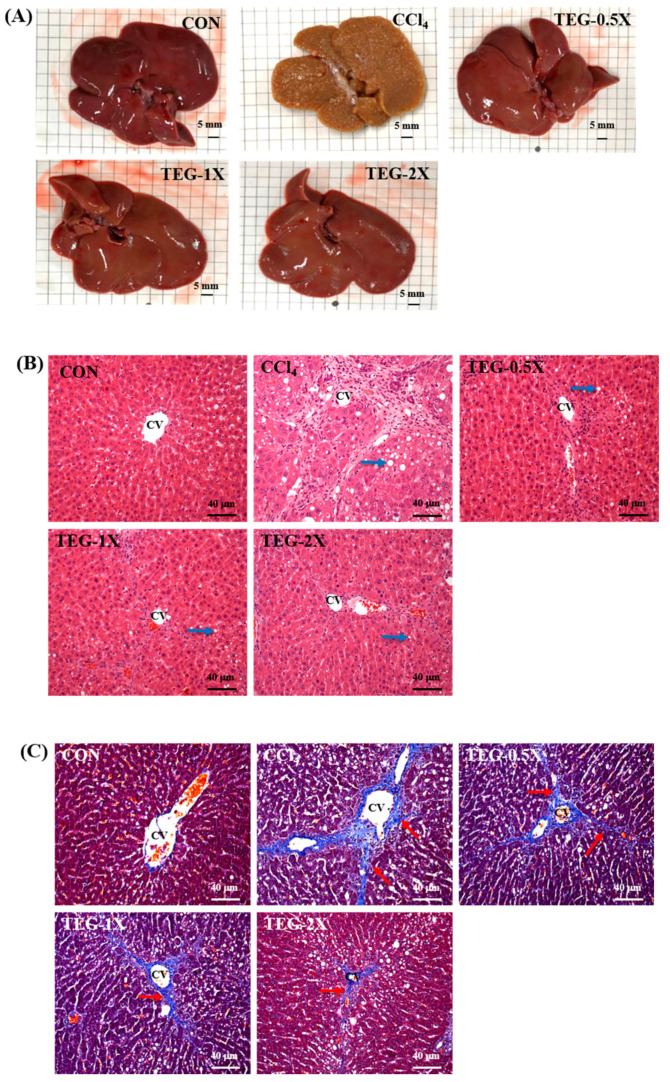
Characteristic livers after traditional essence of ginseng (TEG) supplementation with carbon tetrachloride (CCl_4_)—induced liver damage: (**A**) macroscopic characteristic of livers in CON, CCl_4_, and TEG supplemented groups (CCl_4_, TEG-0.5X, TEG-1X, and TEG-2X) (Scale bar, 5 mm). (**B**) H&E stain of liver tissues in CON, CCl_4_, and TEG supplemented groups (CCl_4_, TEG-0.5X, TEG-1X, and TEG-2X). CV: central vein, blue arrows mark vacuoles caused by steatosis (magnification: ×200; scale bar: 40 μm). (**C**) Masson’s trichrome stain of liver tissues in CON, CCl_4_, and TEG supplemented groups (CCl_4_, TEG-0.5X, TEG-1X, and TEG-2X). CV: central vein, red arrows mark collagen fibers. (magnification: ×200, scale bar: 40 μm).

Macroscopic observation of liver tissues (Figure 5A) in the CCl_4_ group showed a rough liver surface with nodular protrusions of different sizes that were brown in appearance and firm to the touch. In the TEG-0.5X, TEG-1X, and TEG-2X groups, the liver surfaces were smooth with flat nodular protrusions that were dark red in appearance and soft to the touch.

H&E stain histopathological (Figure 5B) examinations showed significant increases in fatty changes, bile duct hyperplasia, inflammatory cell infiltration, necrosis, and fibrosis (*p* < 0.05) in the CCl_4_ group. In contrast to the CCl_4_ group, the 0.5X, 1X, and TEG-2X groups had significant decreases in fatty changes, bile duct hyperplasia, inflammatory cell infiltration, and necrosis (*p* < 0.05). Moreover, the TEG-2X group showed significant decreases in fibrosis (*p* < 0.05) (Table 3). These data indicate that CCl_4_ induced steatosis, necrosis, inflammation, and fibrosis in the rats. However, the supplementation of TEG improved the histology of CCl_4_—treated rat livers, especially in the TEG-2X group for inhibition of fibrosis. The results also showed that the CCl_4_—treated group reflected more collagen fiber, and the phenomenon of accumulation was noted in Masson’s trichrome stain (Figure 5C). However, we found that TEG supplementation reduced the generation and accumulation of collagen fiber.

## 4. Discussion

The results show that the TEG contained a high level of ginsenoside Rg2. After supplementation with TEG for nine consecutive weeks and CCl_4_—induced liver injury for eight weeks, the concentrations of liver injury biochemical indicators [16] in animal serum decreased significantly, and, in liver tissue, the antioxidant activity significantly improved and the accumulation of lipids decreased. Pathological sections showed reduced liver lipid accumulation and fibrosis.

In the CCl_4_—induced chemical liver injury model, AST and ALT are important liver injury markers. In particular, AST, known as the main enzyme in hepatocytes, exists in the blood at a level about 3000-fold higher than that in liver cells when 1% of hepatocytes are damaged [17,18]. Medicinal plants have significant therapeutic value, and plant natural products or extracts can be associated with the infiltration of inflammatory neutrophils and macrophages to counter pathological changes, such as lipid infiltration, autophagy, and apoptosis [19]. Especially in hepatic protection, the medicinal herb *Terminalia belerica* Roxb has been observed to reduce glutathione levels in carbon tetrachloride-affected rats to improve hepatic function [20]. In the traditional system of Chinese medicine, the extracts of *Ginkgo biloba* leaves have been used to protect neurons, and they have also been proven to have a hepatoprotective effect against CCl_4_—induced hepatotoxicity in rats. Their effect is related to the inhibition of lipid peroxidative processes, and they further prevent GSH depletion that begins when *G. biloba* phytosomes exert their antioxidant activity by a two-fold action [21].

Ginseng is also a well-known traditional herb. Previous studies have shown that pre-treatment with *P. ginseng* CA Meyer can reverse liver toxicity induced by benzo[α]pyrene. Elevated plasma ALT and AST levels can be decreased by revised GSH content and glutathione S-transferase activity [22]. Ginsenosides are the major active ingredients of ginseng. They have been reported to have neuroprotective effects on SOD and GPX by preventing lipid peroxidation as a result of oxidative stress [23]. It may be due to the mitochondrial membrane being stable and maintaining the mitochondrial structure, leading to complete retention of mitochondrial functions. During energy respiration and oxidative phosphorylation, numerous electrons leak out from the uncoupled electron transport chain [24,25]. When the reactive oxygen species level is high, free radical scavengers are depleted and attack the antioxidant system, leading to excessive oxidative stress in the body [26]. Therefore, protecting the integrity of liver mitochondria by stabilizing the structure and function is important to maintaining the normal function of cells, resisting reduced ROS formation, and protecting against CCl_4_—induced cytotoxicity [27].

According to previous studies, ischemia-reperfusion might be related to cell apoptosis [28,29]. However, ginsenoside Rg2 can increase cell antioxidants by decreasing lipid peroxidation (e.g., the excessive production of malondialdehyde and nitric oxide) with the protein expression levels of calpain II, caspase3, and beta-amyloid1-40 in PC12 cells with introduced glutamate [30]. Ginsenoside Rg2 inhibits the influx of Na^+^ through the channels by acting on nicotinic acetylcholine receptor-operated cation channels, consequently reducing both the Ca^2+^ influx and catecholamine secretion in chromaffin cells [31]. This process can down-regulate the expression of pro-apoptotic factors BAX and P53 and up-regulate the BCL2 family of proteins and mitochondrial hsp70 (HSP70) to maintain mitochondrial function. These results demonstrate that ginsenoside Rg2 has a neuroprotective effect by preventing cell apoptosis [11,31,32,33]. Liver fibrosis is a physiological response to chronic or iterative liver injury and can progress to cirrhosis over time [34]. Approximately 45% of all cirrhosis deaths are related to fibroproliferative diseases [35]. Extracellular matrix (ECM) turnover and its production affect cell development and lead to complications of cirrhosis by indirect complex matrix biology and provide an early signal to generate collagen [36]. Accumulating studies have indicated that the Nrf2 gene in hepatocytes can regulate the antioxidant and inflammatory response factors that promote liver fibrosis by blocking downstream signaling pathways [37,38,39,40], and the antioxidant activity decreases liver fibrosis [41]. Our research results show that the antioxidant Rg2 in TEG may reduce oxidative stress and keep cell function intact without apoptosis, and, furthermore, reduce the occurrence of fibrosis caused by chemical liver injury.

## 5. Conclusions

The results of this study show that supplementation with 0.5X, 1X, and 2X doses of traditional essence of ginseng with a high content of ginsenoside Rg2 for nine consecutive weeks affected multiple clinical liver-function-related indicators, suggesting that the combination of increasing the antioxidant status, reducing fat accumulation, and inhibiting inflammation can reduce liver damage. This supplementation can help reduce aspartate aminotransferase and alanine aminotransferase in serum, reduce serum triglyceride and total cholesterol, and increase antioxidant status to achieve a protective function. We also observed that the TEG dose dependently inhibited the rise of AST, ALT, TC, and TG, and restored the levels of antioxidant enzymes, i.e., GSH, GPX, GR, SOD, and CAT, as well as TC and TG in liver contents of CCl_4_—treated rats. In conclusion, these results suggest a protective effect of TEG in rats against CCl_4_—induced liver injuries.

## Figures and Tables

**Table 1:** Effects of traditional ginseng essence (TEG) on hepatic antioxidative parameters in rats with carbon tetrachloride (CCl_4_)—induced liver damage at the eighth week.

Group	GSH	GPX	GR	SOD	CAT
	(μM/mg)	(nmol/min/mg)	(nmol/min/mg)	(U/mg)	(nmol/min/mg)
**CON**	1.61 ± 0.10 ^b^	11.68 ± 0.62 ^d^	14.06 ± 0.41 ^b^	0.082 ± 0.023 ^b^	33.08 ± 0.48 ^d^
**CCl_4_**	1.44 ± 0.06 ^a^	8.86 ± 0.27 ^a^	9.18 ± 0.69 ^a^	0.061 ± 0.005 ^a^	28.65 ± 0.32 ^a^
**TEG-0.5X**	1.61 ± 0.08 ^b^	9.71 ± 0.43 ^b^	14.06 ± 0.57 ^b^	0.062 ± 0.004 ^a^	30.87 ± 0.67 ^b^
**TEG-1X**	1.60 ± 0.07 ^b^	9.75 ± 0.25 ^b^	14.15 ± 0.68 ^b^	0.066 ± 0.003 ^a^	30.84 ± 0.32 ^b^
**TEG-2X**	1.62 ± 0.11 ^b^	11.11 ± 0.45 ^c^	14.78 ± 0.49 ^c^	0.079 ± 0.007 ^b^	32.09 ± 0.57 ^c^

Values are presented as mean ± SD for *n* = 8. Values in a column with the same letters (a, b, c, d) are not significantly different at *p* < 0.05 according to one-way ANOVA with Cochran-Armitage test and trend analysis. CON, control group. CCl_4_, CCl_4_ administration only. TEG-0.5X, CCl_4_ administration with 0.5 times the daily recommended dosage of TEG. TEG-1X, CCl_4_ administration with daily recommended dosage of TEG. TEG-2X, CCl_4_ administration with two times the daily recommended dosage of TEG. GSH: glutathione. GPX: glutathione peroxidase. SOD: superoxide dismutase. CAT: catalase. GR: glutathione reductase.

**Table 2:** Absolute weight and relative weight of liver changes in each group of rats after 9 weeks of supplementation with traditional essence of ginseng (TEG) and 8 weeks of carbon tetrachloride (CCl_4_)—induced liver injury.

Group	Liver	Relative Liver
	(g)	(%)
**CON**	13.6 ± 1.2 ^a^	2.80 ± 0.36 ^a^
**CCl_4_**	16.5 ± 2.5 ^c^	3.62 ± 0.56 ^c^
**TEG-0.5X**	16.6 ± 2.0 ^c^	3.57 ± 0.47 ^c^
**TEG-1X**	15.6 ± 2.0 ^bc^	3.28 ± 0.38 ^bc^
**TEG-2X**	14.5 ± 1.4 ^ab^	2.98 ± 0.29 ^ab^

Values are the mean ± SD for *n* = 8. Values in a column with the same letters (a, b, c) did not significantly differ at *p* < 0.05, according to one-way ANOVA with the Cochran−Armitage test and trend analysis. CON, control group. CCl_4_, CCl_4_, administration only. TEG-0.5X, CCl_4_ administration with 0.5 times the daily recommended dosage of TEG. TEG-1X, CCl_4_ administration with daily recommended dosage of TEG. TEG-2X, CCl_4_ administration with two times the daily recommended dosage of TEG.

**Table 3:** Effects of traditional essence of ginseng (TEG) on hepatic histopathology scores in rats with carbon tetrachloride (CCl_4_)—induced liver damage.

Group	Fatty Changes	Bile Duct Hyperplasia	Inflammatory Cell Infiltration	Necrosis	Fibrosis
**CON**	0.00 ± 0.00 ^a^	0.00 ± 0.00 ^a^	0.00 ± 0.00 ^a^	0.00 ± 0.00 ^a^	0.00 ± 0.00 ^a^
**CCl_4_**	2.38 ± 1.06 ^c^	2.00 ± 0.53 ^c^	2.13 ± 0.83 ^c^	1.75 ± 0.71 ^c^	1.63 ± 0.74 ^c^
**TEG-0.5X**	1.13 ± 0.83 ^b^	1.00 ± 0.53 ^b^	1.63 ± 0.74 ^b^	1.13 ± 0.35 ^b^	1.00 ± 0.76 ^bc^
**TEG-1X**	1.13 ± 0.64 ^b^	1.25 ± 0.46 ^b^	1.50 ± 0.76 ^b^	1.00 ± 0.53 ^b^	1.13 ± 0.64 ^bc^
**TEG-2X**	1.38 ± 0.92 ^b^	1.00 ± 0.76 ^b^	1.25 ± 0.46 ^b^	1.13 ± 0.64 ^b^	0.75 ± 0.71 ^b^

Values are presented as mean ± SD for *n* = 8. Values in a column with the same letters (a, b, c) do not significantly differ at *p <* 0.05 according to one-way ANOVA with the Cochran−Armitage test and trend analysis. CON, control group. CCl_4_, CCl_4_, administration only. TEG-0.5X, CCl_4_ administration with 0.5 times the daily recommended dosage of the TEG. TEG-1X, CCl_4_ administration with daily recommended dosage of TEG. TEG-2X, CCl_4_ administration with two times the daily recommended dosage of TEG.
